# First report of DNAI2-associated primary ciliary dyskinesia in Libya: A case from a nonconsanguineous marriage

**DOI:** 10.1016/j.rmcr.2025.102281

**Published:** 2025-09-15

**Authors:** Ziyad Fathi Alatrash, Abdulgader ahmed Elbousify, Ahmed Ibrahim Elbousify

**Affiliations:** a Faculty of Medicine, University of Tripoli, Tripoli, Libya; b Consultant and Head of Respiratory and Allergy Department, University of Tripoli, Libya

**Keywords:** Primary ciliary dyskinesia, DNAI2 gene, Mutation, Cilia, Child, Bronchiectasis

## Abstract

Primary ciliary dyskinesia (PCD) is an autosomal recessive condition resulting from defects in motile cilia structure and function, impairing mucociliary clearance. This defect predisposes such patients to frequent sinopulmonary infections. The chronic productive cough is a key symptom, with progressive bronchiectasis as a common accompaniment, although diagnosis is delayed, with the mean age at diagnosis being about 11 years. Recent studies have indicated that PCD is highly genetically heterogeneous with more than 50 PCD genes identified to date. Herein, we describe an 11-year-old child with clinical and radiologic characteristics of PCD, from a non-consanguineous family, in whom genetic testing indicated a rare homozygous mutation in DNAI2. This case emphasizes the importance of a high level of suspicion of PCD in children with a history of recurrent, unexplained respiratory infections. The ability to diagnose this new disease at the molecular level rapidly is significant for early and specific treatment, and emphasizes the critical importance of a close cooperation between the clinicians and the family for effective care and control of the patient. Our results support inclusion of comprehensive genetic testing in the first tier diagnostic approach to accelerate the time to accurate diagnosis of PCD.

## Highlights

• First reported case of a rare homozygous *DNAI2* mutation in an 11-year-old Libyan boy from a non-consanguineous family suffering from primary ciliary dyskinesia (PCD).
• Emphasizes the importance of multidisciplinary care and close physician-parent cooperation to optimize long-term outcomes for patients with genetically proven PCD.

## 1 Introduction

Primary Ciliary Dyskinesia (PCD) is an inherited ciliopathy that presents as chronic sinopulmonary disease secondary to altered mucociliary clearance. Although it can have a variety of clinical manifestations, it is commonly recognized by typical findings, such as neonatal respiratory distress and situs inversus, seen in ∼50 % of the cases [1]. However, a large proportion of patients are not described by these classic syndromic manifestations, which gives the impression of prolonged diagnostic delay and of irreversibility of lung damage in the absence of bronchiectasis treatment [2].

The genetic bases of PCD is heterogeneous, with pathogenic variants in more than 50 genes reported, among which rare variants of the DNAI2 gene encoding an outer dynein arm component [3]. With a wide clinical expressivity, genetic analysis becomes an essential tool for both diagnosis and to determine cases that may remain undiagnosed. We present a severe case of an 11-year-old female with a confirmed PCD diagnosis by genetic testing with a homozygous mutation in DNAI2 with severe respiratory disease. This report is also important in highlighting the importance of keeping PCD in mind in patients that present with a clinical phenotype suitable for PCD regardless of the absence of situs inversus or a history of consanguinity and therefore the importance of molecular diagnostics in the current clinical pathway. This case report is in line with the SCARE criteria. [4].

## 2 Case presentation

### 2.1 Medical history

At 29–7–2024, an 11-year-old female presented with recurrent emergency department visits due to chronic wet cough, recurrent chest infection, and episodes of dyspnea since birth. Despite multiple courses of treatment, her symptoms persisted, prompting further investigation.

### 2.2 Lab results

**Fig. 1:**
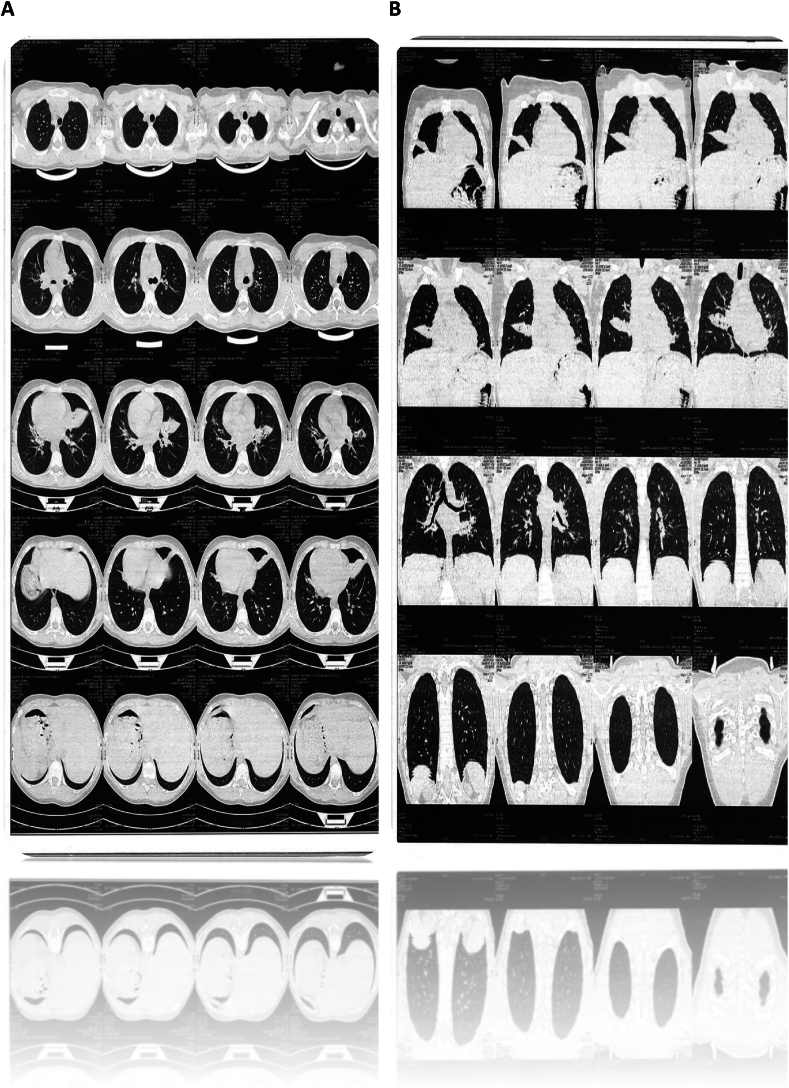
Axial CT scan of the chest plain Axial CT Scan Display a Series of Cross-Sectional Images. In the review of the CT axial Chest CT scan, it reveals the presence of patchy opacities seen in the right middle lobe & band like opacity with mild bronchiectatic changes seen in the lingula Segments of left upper lobe. The opacities were distributed in non-uniform pattern across the multiple CT images.

A chest HRCT was sent which demonstrated bilateral early bronchiectasis, and persistent atelectasis of right middle lobe. CT of paranasal sinuses was ordered which revealed near total opacification of the sinuses with clinical suspicion of PCD [Fig. 1]. In view of her background of recurrent respiratory infections, bronchoscopy with mucosal biopsy was performed and sent for genetic study. Genetic Testing (September 28, 2024): Despite the nonconsanguineous status of her parents, PCD was identified by a homozygous mutation in the DNAI2 gene. This case underlines the necessity of early suspicion, genetic confirmation and a multidisciplinary management in cases of recurring respiratory infections. A DNA extract (from EDTA anticoagulated blood) was available. Whole exome was captured using xGen Exome Research Panel v2 and sequenced on a NovaSeq X (Illumina, San Diego, CA, USA). In total, we brought back 190,610,958 bases of sequence and aligned against the Genome Reference Consortium Human Build 38 (GRCh38) with the average coverage of 138.41. The median coverage depth for the region analyzed was about 20.0, which is in the range of depth often reported for good quality exome sequencing and was deemed adequate for coverage for this region.

**Fig. 2:**
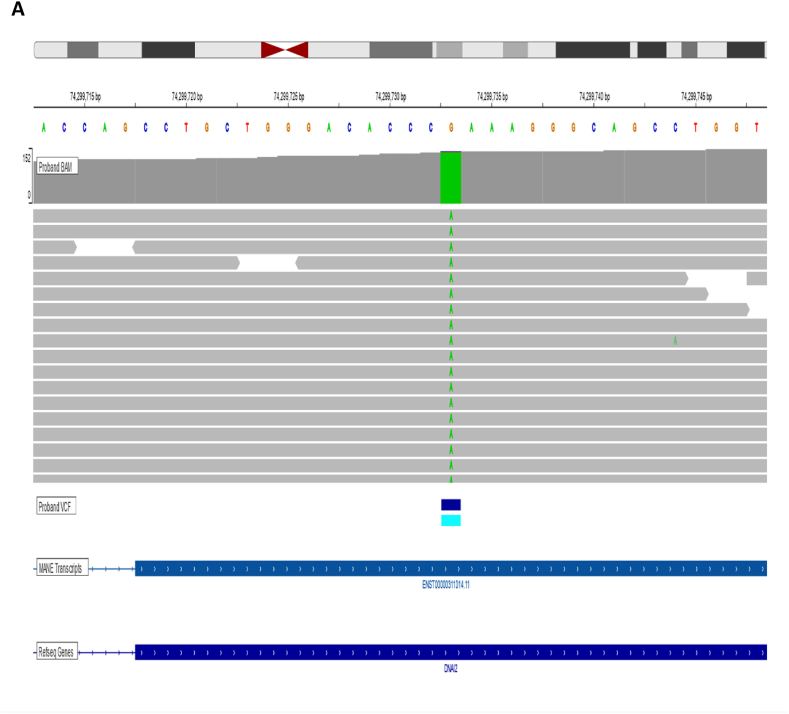
EPH24-SKQN. Identification of NM_023036.6:c.740G>A (p.Arg247Gln) homozygous variant. The BAM file is displayed in The Integrative Genomics Viewer (IGV).

Sequencing information was annotated and interpreted with EVIDENCE v4. 2 based on the American College of Medical Genetics and Genomics and Association for Molecular Pathology guidelines. PCD was diagnosed after homozygous DNAI2 pathogenic variant was detected. The pathogenicity of the variant was determined based on its clinical phenotype with the patient, since the variant is known to cause disruption of the cilia function. Low-confidence variants were validated by Sanger sequencing [Fig. 2].

**Fig. 3:**
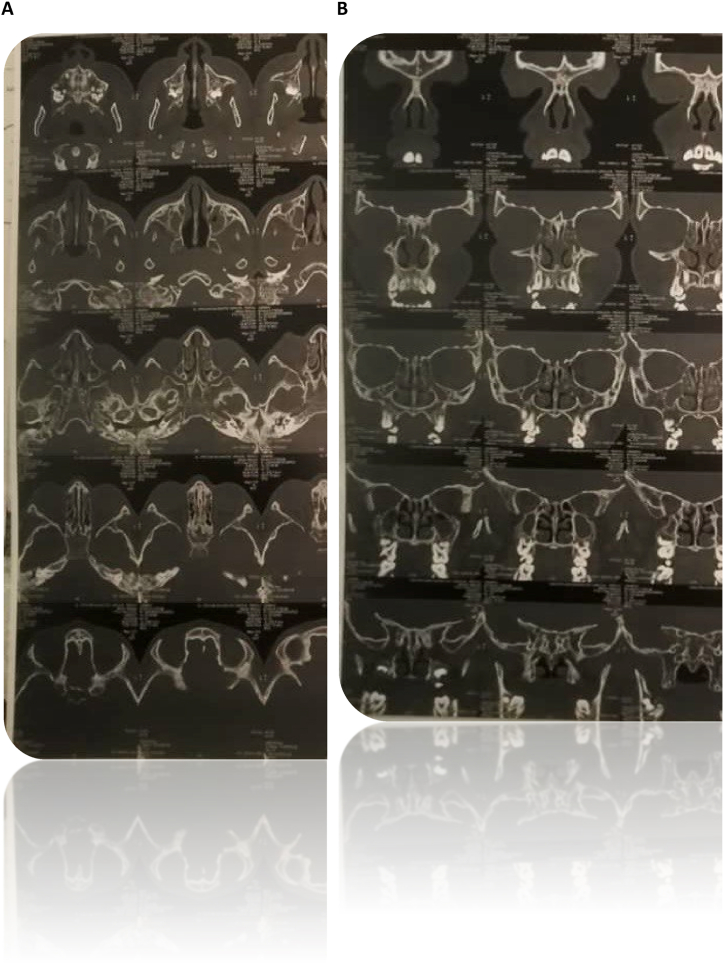
Ct scan of the paranasal sinuses (Axial and coronal views) Plain CT Scan for the Evaluation of Sinus Pathology. In the review of the CT scan of the paranasal sinuses, there is near-total opacification of the maxillary and sphenoidal sinuses, as well as ethmoidal air cells. Notable mucosal thickening and bony border irregularities of the paranasal sinuses are also observed. There is associated opacification of the left middle ear cleft suggestive of otitis media. These radiological features are consistent with chronic pansinusitis. Due to the history of recurrent chest infections and chronic sinusitis, further evaluation to rule out Primary Ciliary Dyskinesia (Immotile Cilia Syndrome) is recommended.

“Fig. 3. CT (Computed Tomography) Scan of the Paranasal Sinuses. (A) Axial view, (B) Coronal view showing near total opacification of maxillary and sphenoidal sinus as well as ethmoidal air cells. There is significance mucosal thickening and bony margin irregularity of the paranasal sinuses. Pseudo-opacification of left middle ear cleft opacification is compatible with the diagnosis of otitis media. Those radiological patterns are suggestive for chronic pansinusitis and are in accordance with the clinical suspicion of Primary Ciliary Dyskinesia."

Treatment and Management: Once PCD was established, a multidisciplinary approach was initiated to treat the patient in a holistic way, including the correction of aberrant ciliary function to regularize MCC and treatment of lung infections. Pharmacological Therapy:

Nebulization strategy: We used budesonide, ipratropium and salbutamol for control of inflammation of the airways and bronchospasm. ––– Antibiotic treatment: The patient was treated with Levofloxacin 750 mg for 2 weeks to remove the infectious source.

∗∗Mucolytics and Accessory: the same here - as formoterol/budesonide and acetylcysteine to aid clearance of mucus and to assist in keeping the airway open.

Supportive Meds: Levocetirizine, Vitamin K for allergic rhinitis. Non-Pharmacological Therapy.

- Chest physiotherapy: The purpose was to move mucus in the airways mechanically. - Sinuses: He was encouraged to use the neti pot daily as well as corticosteroid nasal spray for chronic sinusitis. Monitoring: A second bronchoscopy was scheduled after treatment for treatment adjustment based on bronchus condition.

Results and Follow-Up: After the precise genetic diagnosis and the initiation of a global multidisciplinary management, the clinical course showed improvement over time. 1st follow up (August 5th, 2024) found much less cough and a considerably improved airway cleaning mainly due to targeted medication and effective physiotherapy.

The patient has been subsequently followed within a multidisciplinary evidence based assimilated regimen with monthly follow-up. She is currently clinically stable and asymptomatic, which represents a significant improvement compared to her pre-diagnosis state of chronic respiratory insufficiency. She has currently been living with daily nebulization, regular chest physiotherapy and targeted antibiotic therapy according to clinical and culture findings. Preventative vaccinations and aggressive chronic sinusitis management (including nasal rinse/spray and topical steroids) are also important in her management.

This durable response highlights the potential benefits of precise genetic diagnosis followed by holistic PCD management. It serves to emphasize that early diagnosis and appropriate therapy in such patients can result in very favorable long-term clinical results and thus markedly improved lifestyle. Regular monitoring will be continued for our patient, so as to retain her stable condition and maximize long-term outcome.

## 3 Discussion

Primary Ciliary Dyskinesia (PCD) is a genetically heterogeneous condition due to mutations of cilia causing chronic disease. A delay in diagnosing such patients will assume a significant risk of progressive lung damage, and early and accurate recognition is paramount [5]. In the era of modern diagnostics tools such as high-speed video microscopy and nasal nitric oxide (nNO) diagnostic testing are of the essence, although molecular testing has now become essential for a definitive diagnosis, in particular in atypical examples [6]. The genetic landscape of PCD is complex, with >45 known genes [7]. Classically these present with neonatal respiratory distress and the triad of symptoms described by Kartagener [8], most. situs inversus.

This case report describes an 11-year-old female patient with homozygous mutation in DNAI2, a previously described (but very rare) cause of PCD (2–4 % of patients) that disrupts the ODA complex [9]. However, the scientific value of this case goes beyond the case report of an uncommon mutation. Its major implication is as a potent clinical reminder that PCD should be considered even in the absence of classic syndromic signs.

Our patient had severe recurrent sinopulmonary disease, but did not have the situs inversus, which is often present and triggers early suspicion. This lack may result in substantial diagnostic delays owing to failure of physicians to suspect PCD. In this light, this case represents an important reminder of the variability in expressivity of the condition and the difficulty in relying solely on syndromic patterns to make a diagnosis. Arguably one of the most powerful points it makes is that a patient's clinical phenotype (a history of unexplained y wet cough, bronchiectasis, chronic sinusitis) should be the main motivator for investigating for PCD.

This case also emphasizes the importance of an early genetic testing in the diagnostic pathway and not only as a confirmation when all other signs are there. Here the molecular diagnosis was not only the academic icing on the cake, but a major player that helped to open the door to targeted, multi-disciplinary therapeutic strategy. This resulted in a significant improvement of the patient's clinical condition and therefore quality of life, illustrating the immediate and high effect of an accurate genetic diagnosis for the patient. The event that they were born to a non-consanguineous family further enhances the generalizability of this lesson, highlighting that PCD is a consideration for any child with an appropriate clinical history independent of family structure.

## 4 Conclusion

This case report has made a significant contribution with regards to demonstrating that PCD can present in the absence of its phenotypic, syndromic features, including situs inversus. The confirmatory diagnosis of a non-consanguineous homozygous ∗DNAI2∗ mutation emphasizes the need for a critical clinical message: PCD must be considered based on phenotype alone with a sufficiently high index of clinical suspicion, especially in the scenario of unexplained chronic sinopulmonary disease. This study strongly supports the inclusion of genetic testing upfront as a central diagnostic tool; one of significance, and not simply a supportive one. In doing so, it enables timely, life-changing interventions and extends the clinical spectrum of this rare condition, delivering a unique, actionable insight for clinicians and researchers around the world.

## CRediT authorship contribution statement

**Ziyad Fathi Alatrash:** Writing – review & editing, Writing – original draft, Methodology, Investigation, Formal analysis. **Abdulgader ahmed Elbousify:** Writing – review & editing, Resources, Methodology. **Ahmed Ibrahim Elbousify:** Writing – review & editing, Validation, Supervision, Resources, Project administration, Methodology, Investigation, Data curation, Conceptualization.

## Ethical approval

Ethical approval was obtained on March 3, 2025.

## Consent

Written informed consent was obtained from the patient for publication of this case report and any accompanying images. A copy of the written consent is available for review by the Editor-in-Chief of this journal upon request.

## Reporting guidelines

This work has been reported in line with the SCARE 2023 criteria^4^ [Sohrabi et al., Int J Surg Lond Engl. 2023; 109(5):1136].

## Sources of funding

This study received no funding.

## Funding

None.

## Declaration of competing interest

The authors declare that they have no known competing financial interests or personal relationships that could have appeared to influence the work reported in this paper.
